# Prickly Heat

**Published:** 1899-08-05

**Authors:** 


### PRICKLY HEAT.

Prickly heat is not a very common disease in England; still when it does occur it is sufficiently annoying to make up for its infrequency, and this is just the time of year when it may be looked for. Dr. Frederick Pearce, of Bombay, writing in the *Journal of Tropical Medicine*, maintains that the disease is caused by the increased activity set up in the sebaceous glands in consequence of excessive sweating. In other words, he looks upon prickly heat as an acute seborrhœa, maintained in a more or less active state by the continued irritation of excessive perspiration. On this view he hangs a very important detail as to treatment. All things which increase perspiration are, of course, to be avoided, seeing that in proportion to the flow of perspiration will be the irritation of the sebaceous glands, but the great thing to remember is that the skin is to be kept greasy. Soap should not be used in washing, because it removes the sebaceous material from the skin which, on being freed from its natural oil, tends to become dry, rough, and hard, thus stimulating the sebaceous glands to produce more secretion, while at the same time the excessive perspiration is also irritating them to lubricate the surface. The removal of the natural grease of the skin under these circumstances is distinctly disadvantageous, and bathing should be performed with plain water. Soap, he says, is only required when bathing is neglected. Curative treatment follows upon the same lines. The body should be anointed with oil for the purpose of protecting it against the irritation of the exuded sweat. The best preparation is, perhaps, a mixture of almond oil and lanoline, in the proportion, say, of eight to one, and scented according to fancy. It must be remembered that vaseline is not equivalent to oil for this purpose.

